# Interaction between Cholesteryl Ester Transfer Protein and Hepatic Lipase Encoding Genes and the Risk of Type 2 Diabetes: Results from the Telde Study

**DOI:** 10.1371/journal.pone.0027208

**Published:** 2011-11-03

**Authors:** Laura López-Ríos, Francisco J. Nóvoa, Ricardo Chirino, Francisco Varillas, Mauro Boronat-Cortés, Ana M. Wägner

**Affiliations:** 1 Servicio de Endocrinología, Complejo Hospitalario Universitario Insular Materno-Infantil de Gran Canaria, Las Palmas de Gran Canaria, Spain; 2 Departamento de Ciencias Médicas y Quirúrgicas, Universidad de Las Palmas de Gran Canaria, Las Palmas de Gran Canaria, Spain; 3 Departamento de Bioquímica, Biología Molecular, Fisiología, Genética e Inmunología. Universidad de Las Palmas de Gran Canaria, Las Palmas de Gran Canaria, Spain; 4 Hospital General de Fuerteventura, Fuerteventura, Spain; Roswell Park Cancer Institute, United States of America

## Abstract

**Background and Aim:**

Diabetic dyslipidaemia is common in type 2 diabetes (T2D) and insulin resistance and often precedes the onset of T2D. The Taq1B polymorphism in *CETP* (B1 and B2 alleles) (rs708272) and the G-250A polymorphism in *LIPC* (rs2070895) are associated with changes in enzyme activity and lipid concentrations. Our aim was to assess the effects of both polymorphisms on the risk of T2D.

**Methods and Results:**

In a case-control study from the population-based Telde cohort, both polymorphisms were analysed by PCR-based methods. Subjects were classified, according to an oral glucose tolerance test, into diabetic (N = 115) and pre-diabetic (N = 116); 226 subjects with normal glucose tolerance, matched for age and gender, were included as controls. Chi-square (comparison between groups) and logistic regression (identification of independent effects) were used for analysis. The B1B1 Taq1B *CETP* genotype frequency increased with worsening glucose metabolism (42.5%, 46.1% and 54.3% in control, IGR and diabetic group; p = 0.042). This polymorphism was independently associated with an increased risk of diabetes (OR: 1.828; IC 95%: 1.12–2.99; p = 0.016), even after adjusting by confounding variables, whereas the *LIPC* polymorphism was not. Regarding the interaction between both polymorphisms, in the B1B1 genotype carriers, the absence of the minor (A) allele of the *LIPC* polymorphism increased the risk of having diabetes.

**Conclusion:**

The presence of the B1B1 Taq1B *CETP* genotype contributes to the presence of diabetes, independently of age, sex, BMI and waist. However, among carriers of B1B1, the presence of GG genotype of the -250 *LIPC* polymorphism increases this risk further.

## Introduction

Diabetic dyslipidaemia is characterised by hypertriglyceridaemia, low high-density lipoprotein (HDL) cholesterol (c) and normal low-density lipoprotein-cholesterol (LDLc) but preponderance of small-dense, highly atherogenic particles. The increase in free fatty acids (FFAs) as degradation products of triglycerides (TGs) is associated with the development of insulin resistance [1].

Cholesteryl Ester Transfer Protein (CETP) and Hepatic Lipase (HL) are central enzymes in the metabolism of HDL particles and reverse cholesterol transport. CETP is responsible for an exchange of cholesteryl ester (CE) for triglycerides (TGs) between LDL and HDL and TG rich-lipoprotein particles [2]. The result is an enrichment of HDL and LDL particles in TGs, which makes them good substrates for HL [3]. The latter catalyses the hydrolysis of the TGs and phospholipids present in several lipoprotein subclasses, leading to changes in the size and density of lipoproteins [3]. The increased activity of either enzyme results in lower HDLc levels and a predominance of small, dense HDL and LDL particles [4], [5].

The variations in the *CETP* gene, which lead to changes in enzyme function, have consequences on lipoprotein composition. *CETP* deficiency in humans is characterized by increases in HDLc, whereas increases in its activity are associated with an enrichment of HDL particles in TGs and a decrease in HDLc levels [6]. The most extensively studied polymorphism in *CETP* is Taq1B (rs708272) [7]. The G allele, also called B1, is associated with higher enzymatic activity, higher CETP mass and lower HDLc levels [8], [9]. It has been estimated that this polymorphism is responsible for 5.8% of the variation in HDLc levels [10].

Studies in transgenic mice demonstrate that the over-expression of the gene encoding HL, *Lipc*, leads to a marked decrease in plasma HDLc levels[11] , an observation supported by human studies showing an inverse correlation between HL activity and HDLc concentrations [12]. The -G250A *LIPC* polymorphism (rs2070895) [13], located in the promoter region of the gene, has been extensively studied in relation to enzyme activity and lipid metabolism. The minor allele (A) is associated with a reduction of transcriptional activity *in vitro* [14] and a 15–45% reduction in enzymatic activity [15]. In humans, the minor allele has also been associated with an increased HDLc concentration and more buoyant LDL particles [15], [16]. Studies assessing the association of this variant with T2D show conflicting results [17].

Diabetes is often preceded and even predicted, by the presence of dyslipidemia [13]. Thus, mechanisms involved in the development of diabetic dyslipidemia may also play a role in the pathogenesis of T2D. The effects of the mentioned polymorphisms in *CETP* and *LIPC* on HDLc concentrations are well established, but their relation with the risk of T2D is less known. Therefore, the aim of our study was to analyze the relationship between polymorphisms in these two genes and the presence of diabetes and insulin resistance in a Canarian population.

## Methods

### Study population

The Telde study is a cross-sectional population-based study on the prevalence of diabetes and cardiovascular risk factors in Telde, a city located on the island of Gran Canaria, Spain. The study population and design of this survey has been previously described [18]. An oral glucose tolerance test (OGTT) was performed and the subjects were classified (using ADA 1997 criteria) as diabetic (n = 115) and pre-diabetic (n = 116) if they had impaired fasting glucose, impaired glucose tolerance or both. A total of 226 subjects with a normal OGTT were selected, after matching for gender and age with the other two groups. All participants gave their written informed consent for participation in the study, which was carried out according to the declaration of Helsinki and approved by the local ethics committee.

### Genetic analyses

**Figure 1:**
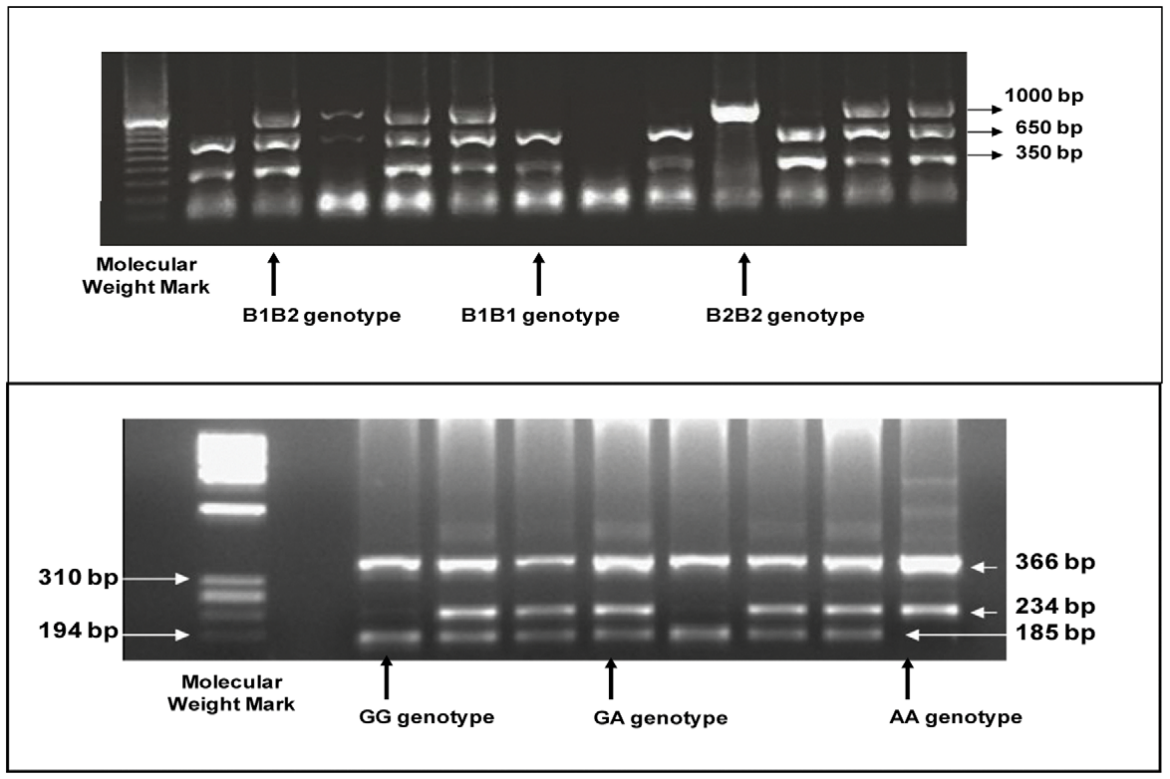
Agarose gel results of both polymorphisms. PCR-RFLP agarose gel (A) after digestion with Taq1B enzyme. The 1000 bp band corresponds to the B2 allele and the 650 and 350 bp bands correspond to the B1 allele. Results of *LIPC* genotyping by ARMS-PCR (B). The 366 bp band is the product of the outer primers, the 234 bp band, of an outer primer and the inner primer for allele A and the 185 bp band, of the other outer primer and the inner primer for the G allele.

The biochemical analyses and insulin resistance parameters have been described previously [19]. Genomic DNA was extracted from whole blood (n = 457) using a salting-out method. The Taq1B *CETP* polymorphism was amplified by PCR-RFLP as described by June Hsieh Wu [20] and the *G-250A LIPC* polymorphism was analyzed by AMRS-PCR (Amplification Refractory Mutation System-Polymerase Chain Reaction) [21]. Two pairs of primers were used, one which amplifies a fragment of 366 bp, common to both alleles (outer primers: 5′-CTT TTC TTT TTC TTT GGG CTT AGG CT-3′ and 5′-AAG ACT GCC CAT TAA TAA TTA ACC TCT CAA-3′) and another pair specific for the SNP (inner primers): 5′-CAA GGT CAG AGT TCC AAA TTA ATC CAC-3′ for the G allele and 5′-TTC CAA ACA CAA CAC AGT AGC TTT CAA-3′ for the A allele. The primers were designed *“in silico”* in a free access web (http://cedar.genetics.soton.ac.uk, accessed in August 2007) and then checked for specificity (http://blast.ncbi.nlm.nih.gov/Blast.cgi, accessed in August 2007). The PCR reaction was carried out in a total volume of 25 µl containing 1∶5 ratio of outer to inner primer concentration. The annealing temperatures were 70°C during 15 sec for the outer primers and 58°C during 25 sec for the inner primers with a 30 sec extension at 72°C. PCR products were mixed with 2 µl of loading buffer and run on 2.5% agarose gel stained with Ethidium Bromide. This resulted in 3 DNA fragments: one of 366 bp, one of 234 bp for the A allele and one of 185 bp for the G allele (figure 1).

### Statistical analysis

Statistical analyses were performed with SPSS for WINDOWS, version 13 (SPSS Inc., Chicago, IL). The quantitative variables are described as mean ± standard deviation (S.D). Before further analyses, variable distribution was checked with the Kolmogorov–Smirnov test. A logarithmic transformation was performed for variables not following a Gaussian distribution. Differences between groups were analyzed using either analysis of variance or analysis of covariance, both with the Bonferroni post hoc correction test, after adjusting for age, gender, Body Mass Index (BMI) and waist. The categorical variables were compared using Fisher's exact test for 2×2 tables and chi-squared or the Mantel-Haenszel test for linear association. The independent contribution of each polymorphism to DM2 risk was analyzed by a multinomial logistic regression model, which included age, gender, BMI and waist. All tests were considered significant if p was <0.05.

Regarding the effect of the interaction of both polymorphisms on the risk of diabetes, the reference category was defined by the non-B1B1 genotype, regardless of the -250G/A *LIPC* genotypes (nonB1B1*CETP* genotype). A second group included B1B1 and non-GG genotypes (B1B1CETP/non-GGLIPC) and a third, B1B1 CETP and *LIPC* GG genotypes (B1B1CETP/GGLIPC).

## Results

### Patient description

The anthropometric, clinical and genetic characteristics of the whole population and their classification according to the OGTT are shown in table 1. The frequencies of the B1B1, B1B2 and B2B2 genotypes of the Taq1B *CETP* polymorphism in the whole population were 46.38%, 41.57% and 12.03% respectively, while the frequencies of GG, AG and AA genotypes of the -250G/A *LIPC* polymorphism were 49.23% 43.54% and 7.22%, respectively The distribution and the allele frequency of both polymorphisms followed the Hardy-Weinberg equilibrium. Due to very low frequencies of the 2 genotypes the B2B2 of the Taq1B *CETP* and the AA of the -250G/A *LIPC* were analyzed in the same category as the corresponding heterozygotic genotype, namely as non-B1B1 (B2 carriers) and non-GG (A carriers), respectively.

**Table 1:** Main features of the study population.

	WHOLE POPULATION (n = 457)	CONTROLS (n = 226)	PRE-DIABETIC (n = 116)	DIABETIC (n = 115)
	Mean ± S.D	Mean ± S.D	Mean ± S.D	Mean ± S.D
Age (y)	55.02±11.09	54.48±11.86	52.25±12.36	58.84±10,60
Sex (male/female)	226/234	104/124	57/59	65/51
BMI (kg/m2)	29.62±5.00	28.60±4.45	30.30±5.03	30.97±5.44
Cholesterol (mmol/L)	5.65±1.02	5.65±1.00	5.64±0.91	5.66±1.16
HDL-C (mmol/L)	1.38±0.32	1.45±0.32	1.35±0.30	1.29±0.31
LDL-C (mmol/L)	3.55±0.87	3.58±0.87	3.53±0.79	3.53±0.96
TG (mmol/L)	1.55±0.91	1.34±0.69	1.65±0.91	1.84±1.18
HbA1c (%)	5.76±4.89	5.51±6.76	5.12±0.45	6.90±1.80
Fasting glucose (mmol/L)	6.04±2.29	4.92±0.52	5.64±0.66	8.66±3.22
Glucose after OGTT (mmol/L)	7.13±2.78	5.50±1.21	8.28±1.76	12.11±3.22
Insulin (pmol/L)	75.4±65.3	59.7±38.7	76.7±45.7	104.9±103.1
HOMA	3.19±4.17	1.91±1.32	2.75±1.61	5.71±5.70
*CETP* Allele B1	67%	65%	67%	71%
Allele B2	33%	35%	33%	29%
LIPC Allele A	29%	30%	28%	26%
Allele G	71%	70%	72%	74%

Continuous variables are expressed as mean and S.D. Allele frequencies are expressed as percentages.

### The polymorphisms and the biochemical variables

In the whole population, the B1B1 genotype carriers showed significantly lower HDLc concentrations than the B2-allele carriers (1.33±0.30 mmol/L vs. 1.44±0.33 mmol/L, p<0.001), as well as higher glucose levels after the OGTT. In the control population, there was a significant difference in HDLc levels (1.38±0.31 mmol/L vs. 1.50±0.31 mmol/L, p = 0.004; B1B1 vs. B2-carriers, respectively) and an almost significant difference in glucose levels after the OGTT (5.64±1.08 mmol/L vs. 5.39±1.29 mmol/L, p = 0.069). There were no significant differences between the groups in the pre-diabetic and diabetic subjects (data not shown).

Regarding the *LIPC* polymorphism, the GG genotype carriers showed significantly higher fasting glucose concentrations than the A allele carriers (non-GG genotype) in the whole population (6.30±2.67 mmol/L vs. 5.79±1.83 mmol/L, p = 0.02). However, only the diabetic subjects showed significantly different glucose levels after the OGTT depending on their genotype (13.03±2.72 mmol/L vs. 11.11±2.94 mmol/L, p = 0.025, respectively).

Given the fact that the HOMA index is not a reliable estimation of insulin resistance in diabetic subjects, this subgroup was not analyzed separately in the analysis of variance or covariance. The groups analyzed were: (1) the whole population, (2) the whole population without diabetes (healthy subjects and pre-diabetic subjects) and (3) the healthy control group. Table 2 displays the main results obtained. In summary, B1B1 carriers of the *CETP* polymorphism showed lower HDLc levels and higher HOMA and post-OGTT glucose in all groups and higher insulin levels in the whole population even after adjusting for confounding variables. On the other hand, the GG genotype carriers of the *LIPC* polymorphism showed higher fasting glucose levels than A-allele carriers.

**Table 2:** The Effect of Taq 1B *CETP* (A) and –G250A *LIPC* (B) polymorphisms on several glucose homeostatic parameters for each population studied.

	A)	WHOLE POPULATION		CONTROLS		NON-DIABETIC POPULATION	
Variables	CETP	Mean (IC95%)	P	Mean (IC95%)	P	Mean (IC95%)	P
FastingGlucose	NonB1B1	5.64 (5.45–5.85)		4.86 (4.78–4.95)		5.08 (4.99–5.17)	
(mmol/L)	B1B1	5.91 (5.69–6.13)	0.079	4.95 (4.85–5.05)	ns	5.19 (5.08–5.29)	ns
Glucoseafter	NonB1B1	6.38 (6.08–6.68)		5.18 (4.98–5.39)		5.92 (5.68–6.17)	
OGTT(mmol/L	B1B1	6.94 (6.60–7.31)	0.017	5.56 (5.31–5.83)	0.023	6.37 (6.08–6.68)	0.021
Insulin	NonB1B1	56.7 (52.7–61.0)		46.7 (42.7–50.9)		51.8 (48.0–55.8)	
(pmol/L)	B1B1	63.2 (58.5–68.4)	0.047	50.9 (48.2–59.5)	0.052	57.5 (52.8–62.6)	0.068
HOMA	NonB1B1	2.05 (1.51–2.23)		1.45 (1.32–1.60)		1.68 (1.55–1.82)	
	B1B1	2.39 (2.18–2.62)	0.017	1.69 (1.51–1.89)	0.038	1.90 (1.74–2.08)	0.039
HDL-C	NonB1B1	1.39 (1.36–1.43)		1.46 (1.41–1.52)		1.42 (1.38–1.47)	
(mmol/L)	B1B1	1.30 (1.26–1.33)	0.000	1.33 (1.28–1.39)	0.002	1.31 (1.27–1.36)	0.001

	B)	WHOLE POPULATION		CONTROLS		NON–DIABETIC POPULATION	
Variables	*LIPC*	Mean (IC95%)	P	Mean (IC95%)	P	Mean (IC95%)	P
FastingGlucose	Non-GG	5.62 (5.42–5.82)		4.88 (4.79–4.98)		5.06 (4.97–5.16)	
(mmol/L)	GG	5.92 (5.71–6.14)	0.044	4.92 (4.83–5.02)	ns	5.19 (5.09–5.29)	0.067
Glucoseafter	Non-GG	6.67 (6.35–7.00)		5.33 (5.11–5.55)		6.08 (5.82–6.35)	
OGOTT(mmol/L)	GG	6.61 (6.28–6.95)	ns	5.36 (5.13–5.60)	ns	6.19(5.92–6.47)	ns
Insulin	Non-GG	58.9 (54.6–63.5)		47.9 (43.6–52.7)		51.8 (47.9–56.0)	
(pmol/L)	GG	60.3 (55.8–65.1)	ns	51.5 (46.6–56.9)	ns	56.8 (52.4–61.5)	ns
HOMA	Non-GG	2.12 (1.93–2.31)		1.49 (1.35–1.65)		1.67 (1.54–1.82)	
	GG	2.28 (2.08–2.50)	ns	1.62 (1.46–1.80)	ns	1.88 (1.73–2.05)	0.055
HDL-C	Non-GG	1.35 (1.31–1.39)		1.41 (1.36–1.47)		1.38 (1.33–1.42)	ns
(mmol/L)	GG	1.35 (1.31–1.39)	ns	1.40 (1.34–1.46)	ns	1.37 (1.33–1.42)	ns

P values from analysis of covariance with the Bonferroni post hoc test, after adjusting for age, gender, Body Mass Index (BMI) and waist.

### Association between CETP and LIPC polymorphisms and the prevalence of type 2 diabetes

**Figure 2:**
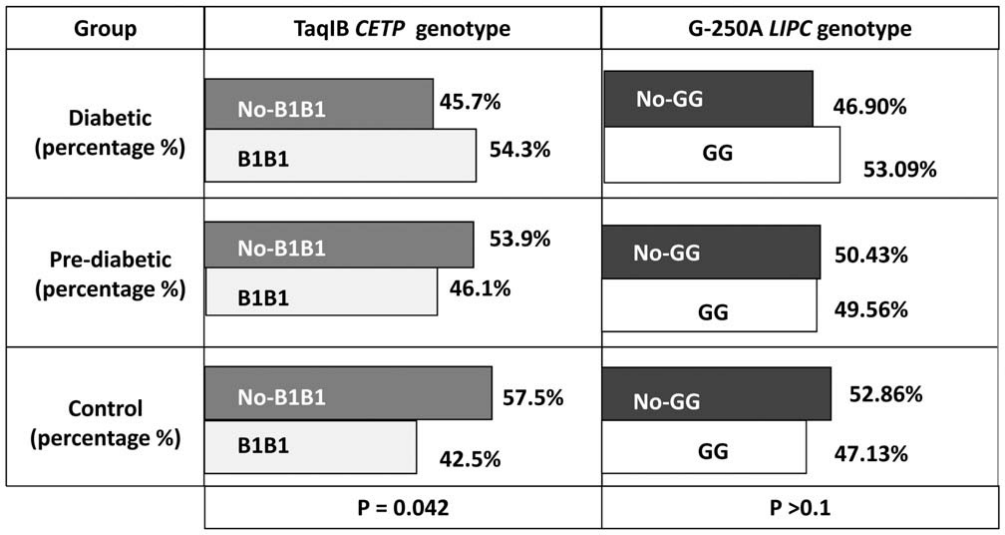
Distribution of Taq1B *CETP* and –G250A *LIPC* genotypes according to OGTT categories. OGTT categories are T2D: type 2 diabetes, pre-diabetes, which includes impaired fasting glucose concentrations, impaired glucose tolerance or both, and healthy controls.

Figure 2 shows the frequency of each polymorphism for the different categories of glucose tolerance (diabetic, pre-diabetic and control groups). The frequency of the B1B1 genotype of *CETP* increased with worsening glucose metabolism, whereas the GG genotype of *LIPC* did not show a significant difference, although a similar trend was found.

The independent effect of each polymorphism on the prevalence of diabetes was assessed using a multinomial logistic regression model adjusting for age, gender, BMI and waist. The B1B1 genotype was associated with an increased risk of diabetes (OR (IC95%): 1.81 (1.12–2.91); p = 0.002), but not pre-diabetes (OR (IC95%): 1.11 (0.70–1.77); p = ns). On its own, the *LIPC* polymorphism was not significantly associated with the risk of diabetes. Finally, we analyzed the effect of the interaction of both polymorphisms on the risk of diabetes. We observed that among the B1B1 *CETP* carriers, the presence of the GG *LIPC* genotype increased the risk of having the disease (table 3).

**Table 3:** Multinomial logistic regression model assessing the combined effects of *CETP* and *LIPC* genotypes on the risk of T2D.

VARIABLE	P	O.R	95% CI
NonB1B1 *CETP*		1(ref.)	
B1B1 *CETP*/nonGG *LIPC*		1.32	0.71–2.44
B1B1 *CETP*/GG *LIPC*	0.036	2.42	1.30–4.50

OR: odds ratio, 95% CI: 95% confidence interval.

## Discussion

The roles of CETP and HL on lipid metabolism and reverse cholesterol transport have been extensively described [5]. In this study, we investigated a variant present on each gene encoding these enzymes in relation to the risk of diabetes in a Canarian population. Our results show that the B1B1 genotype of the Taq1B *CETP* polymorphism is associated with more insulin resistance, higher post-OGTT glucose levels and an increased risk of T2D. On other hand, the -250G/A *LIPC* polymorphism is associated with higher fasting glucose levels, but does not seem to confer a risk of T2D by itself. However, the interaction between both polymorphisms does have an effect on the risk of diabetes.

### The Taq 1B CETP polymorphism (rs708272)

Previous studies have shown that the Taq1B *CETP* polymorphism is associated with increased enzyme activity, TG-enriched LDL and HDL particles and low HDLc levels [9], [22]. Besides, high CETP activity has been demonstrated in obese and diabetic subjects [23], [24]. Previous studies have also shown an association between this polymorphism and the metabolic syndrome [25], independently of the well-known effect on HDLc concentrations and insulin resistance [26], a fact that suggests a possible role of CETP on glucose metabolism. However, to our knowledge, this is the first study to show an association between this polymorphism in *CETP* and the risk of T2D. The frequency of the B1B1 genotype increases with worsening glucose tolerance (figure 2) and its presence is associated with the risk of diabetes even after adjusting for other confounding factors such as age, BMI, waist and TG. In addition, we found lower HDLc levels in B1B1 genotype carriers, as well as higher insulin, HOMA and post-OGTT glucose in non-diabetic B1B1 carriers, further supporting its role in the development of T2D. Previously, we proposed that the contribution of the Taq1B *CETP* polymorphism on insulin resistance could be mediated by an increased flux of FFAs from HDL particles to the liver. Since homozygotes for the B1 allele have an increased CETP activity, they should have an increased TG content in their HDL particles, which in turn would become a good substrate for HL. Thus, individuals with the B1B1 genotype would have an increased flux of free fatty acids to the liver from HDL that would decrease the hepatic sensitivity to insulin [26].

### The –G250A LIPC polymorphism (rs2070895)

HL catalyzes the hydrolysis of TG and phospholipids in TG-enriched HDL and LDL particles, giving rise to smaller, denser particles[27]. In fact, HL activity shows an inverse correlation with HDLc concentration. Two (SNPs) have been described in the promoter region of the gene (-514 C<T, rs1800588 and -250G>A, rs2070895) [28], which are in almost complete linkage disequilibrium. The minor allele in the -250A/G polymorphism is associated with low HL activity [29], an increased HDLc concentration[11] and more buoyant LDL particles. In fact, the effect abdominal obesity has on HL activity is cushioned by this allele [30]. Its frequency in our population was similar to that found by others [13], [17], [31]. However, unlike other authors [17], [32], we did not find an influence of the *LIPC* genotype on HDLc concentrations, nor on the risk of T2D, but it was associated with higher glucose concentrations. The latter followed the same direction as the results from the Finnish Diabetes Prevention Study [13], which showed that the GG genotype doubles the risk of progression to T2D. On the other hand, a large Danish cross-sectional study, which included 3082 cases and 4882 controls, was negative in this aspect [33].

### Taq 1B CETP and –G250A LIPC polymorphisms

The epistatic effect of *CETP* and *LIPC* on HDLc concentrations [34], [35] and atherosclerosis[35] has been previously reported. The authors observed a marked increase in HDLc levels in carriers of both minor frequency genotypes [35]. However, to our knowledge, this is the first time the effect of genetic interaction between *CETP* and *LIPC* is assessed on the risk of T2D. Since the minor allele at -250G/A *LIPC* is associated with a decrease in HL activity and a reduction of TG catabolism from HDL and LDL and the TG content in HDL and LDL is, to a certain extent, the result of an increased CETP activity associated to the B1B1 genotype, we propose that the presence of the A allele of -250G/A *LIPC* reduced the risk of T2D among B1B1 Taq1B *CETP* genotype carriers, although the *LIPC* polymorphism was by itself not associated with T2D.

One of the strengths of this study is its population-based design including well-characterized subjects diagnosed using an OGTT [23], [24]. In addition, to our knowledge, this is the first time the effects of *CETP* and *LIPC* are assessed in relation to the risk of T2D. We are also aware of some limitations of the study. Its cross-sectional nature does not allow us to infer causality. Furthermore, our failure to identify an effect of the *LIPC* polymorphism on diabetes risk might be due to sample size, as is suggested by its association with glycemia and its significant interaction with *CETP*. However, the effects we did find are likely to be true positive, biologically plausible effects, with stringent corrections for multiple analyses. We suggest that while the risk of T2D associated to the B1B1 *CETP* genotype is a consequence of hypertriglyceridaemia by the increase of the flux of FFAs from HDL and LDL particles to the liver, a decrease activity of HL associated to -250 A *LIPC* allele reduces that flux of FFAs, and the risk of T2D amongst B1B1 carriers.

In summary, the Taq1B *CETP* polymorphism was significantly associated with HDLc levels and the presence of T2D and, although we did not find the same association between the -250A/G *LIPC* polymorphism and HDLc levels or T2D, the presence of the A allele appears to exert a protective effect in B1B1 genotype carriers in our population. Nevertheless, larger studies performed in different populations are needed to confirm our findings.
